# Accuracy and Comparison of Core Needle Biopsy Diagnoses With Excision Specimen for Diagnosing Fibroepithelial Lesions of the Breast

**DOI:** 10.7759/cureus.64997

**Published:** 2024-07-20

**Authors:** Muhammad Usman Tariq, Alka Rani, Naila Kayani, Abida K Sattar, Lubna Vohra, Romana Idress

**Affiliations:** 1 Histopathology, Al Hada Armed Forces Hospital, Taif, SAU; 2 Histopathology, Aga Khan University Hospital, Karachi, PAK; 3 Surgery, Aga Khan University Hospital, Karachi, PAK

**Keywords:** accuracy, core needle biopsy, phyllodes tumor, fibroadenoma, fibroepithelial lesion

## Abstract

Background

Core needle biopsy (CNB) for fibroepithelial lesions (FELs) of the breast is commonly encountered by histopathologists. The distinction between fibroadenoma (FA) and phyllodes tumor (PT) can be challenging due to overlapping histological features and the limited nature of CNB material.

Objective

This study aimed to assess the accuracy of CNB diagnosis of FA and PT by comparing it with a diagnosis on subsequent surgical excision specimen.

Materials and methods

A total of 166 cases of FELs of the breast who underwent CNB and subsequent surgical excision between January 2001 and December 2020 were included in our study. All microscopy glass slides were reviewed, and diagnosis confirmed.

Results

While 125 (75%) cases based on CNB received a definitive diagnosis of either fibroadenoma or PT, the remaining 41 (25%) cases were better classified on excision specimens and were descriptively diagnosed as fibroepithelial lesions on CNB. Diagnoses on CNB and on subsequent excision specimens were concordant in 113 (90.4%) cases. Among 12 cases that were discordant, three cases diagnosed as FA on CNB were upgraded to PT on excision specimens. Nine cases diagnosed as PT on CNB were diagnosed as FA on excision specimens. These included conventional, cellular, juvenile, and complex FA types. Three PTs, which were reported as FA on CNB, measured 6, 12.5, and 17.5 cm in the greatest dimension. Among 23 cases of PT which were further categorized on CNB, tumor categories changed on excision specimens in three cases. The diagnostic accuracy of CNB diagnosis was 90.4%.

Conclusion

CNB diagnosis showed good accuracy. PT diagnosis should be strongly considered in all tumors measuring >5 cm, especially those exceeding 10 cm. Cellular, juvenile, and complex FAs can be misdiagnosed as PT on CNB. Correlation with clinical and radiological findings can be helpful in establishing correct diagnosis.

## Introduction

Fibroadenoma (FA) and phyllodes tumor (PT) are fibroepithelial lesions (FELs) with different clinical behaviors; hence, the distinction between these two entities is essential. FA has a tendency to regress with time or remain stable, while PT continuously grows and bears the potential of recurrence and even metastasis in malignant cases [1,2]. Due to the ease of performing core needle biopsy (CNB) and its higher sensitivity and specificity for breast carcinoma, it is a routine procedure in the assessment of breast lesions [1,3]. It combines the results of clinical, radiological, and histological assessment and is considered the method of choice for triple assessment of breast lesions [3]. The diagnostic utility of CNB for FELs is limited due to the morphological features of the lesions [1,3]. Although histological features of FA and PT are described in detail in the literature, differentiating between these FELs remains a challenge for histopathologists. Both these lesions have overlapping morphological features and lack definitive cut-off values, making the diagnostic assessment prone to subjectivity [4-6]. The histological features assessed for diagnosing FELs include stromal cellularity, stromal overgrowth, stromal heterogeneity, stromal condensation around breast ducts, intracanalicular and/or pericanalicular growth pattern, atypia, mitotic count per 10 high power fields (HPFs), and fragmentation of the CNB material [1,7]. Assessment of these histological features is limited using a CNB specimen. Therefore, the distinction between FA and benign PT (BPT) is difficult, resulting in the use of equivocal diagnostic terminology “FEL”[1]. Borderline and malignant PT can still demonstrate specific histological features allowing histopathologists to accurately diagnose PT using CNB [7]. A few studies analyzed cases diagnosed equivocally as FEL using CNB with outcomes on the excision specimen [1,6].

This study aimed to assess the accuracy of CNB diagnosis of FA and PT by comparing it with a diagnosis on the subsequent surgical excision specimen. Additionally, we discussed the features that could be helpful in making the correct diagnosis based on CNB in the literature review.

This article was previously presented at the 110th United States and Canadian Academy of Pathologists (USCAP) Annual Meeting, held on March 13-18, 2021, and its abstract was published [8].

## Materials and methods

This was a retrospective cross-sectional study. The study was performed in the Histopathology section, Department of Pathology and Laboratory Medicine, Aga Khan University Hospital, Karachi. The study population comprised patients diagnosed with "fibroadenoma,” phyllodes tumor,” and “fibroepithelial lesion” on CNB, followed by surgical excision between January 2001 and December 2020. A total of 166 patients were included through non-probability consecutive sampling.

Inclusion criteria were patients (irrespective of age and gender) diagnosed with FA, PT, and FEL on CNB with subsequent excision specimens. Exclusion criteria were poor quality or unavailable slides and blocks for histopathological review. 

Data regarding the patient's age, gender, and tumor size were recorded from histopathology reports. Microscopy glass slides of CNB and excision specimens were reviewed by two histopathologists to confirm the diagnosis. The diagnosis using excision biopsy was considered the gold standard.

Mean and median were calculated for the patient's age and tumor size. Frequency and percentage were calculated for diagnoses based on CNB and surgical excision. Accuracy of CNB diagnosis was calculated as the number of CNB diagnoses concordant with surgical excision diagnoses divided by the total number of cases with a definitive diagnosis of "fibroadenoma" or "phyllodes tumor" based on CNB.

Since the study was conducted by reviewing archived material and no identifiable patient information was included, the study was exempted from approval by the institute’s ethics review committee (exemption letter number: 2023-6988-24562).

## Results

All patients were females. From a total of 166 cases, 125 (75%) were reported as a definitive diagnosis on CNB, and 41 (25%) cases were reported as FELs/biphasic lesions without a clear distinction between FA and PT. On CNB, 76 and 49 cases were diagnosed as FA and PT, respectively, while 110 and 56 cases were diagnosed as FA and PT on excision specimens, respectively. Table 1 presents the correlation of CNB-based diagnosis with surgical excision specimen-based diagnosis.

**Table 1:** Correlation between the diagnoses rendered on CNB and excision specimen. (n=166) CNB: Core needle biopsy

CNB diagnosis	Surgical excision diagnosis	
	Fibroadenoma (n=110)	Phyllodes Tumor (n=56)
Fibroadenoma (n=76)	73 (96%)	3 (4%)
Phyllodes Tumor (n=49)	9 (18.4%)	40 (81.6%)
Fibroepithelial lesion, spindle cell lesion and descriptive diagnosis (n=41)	28 (61%)	13 (39%)

**Figure 1:**
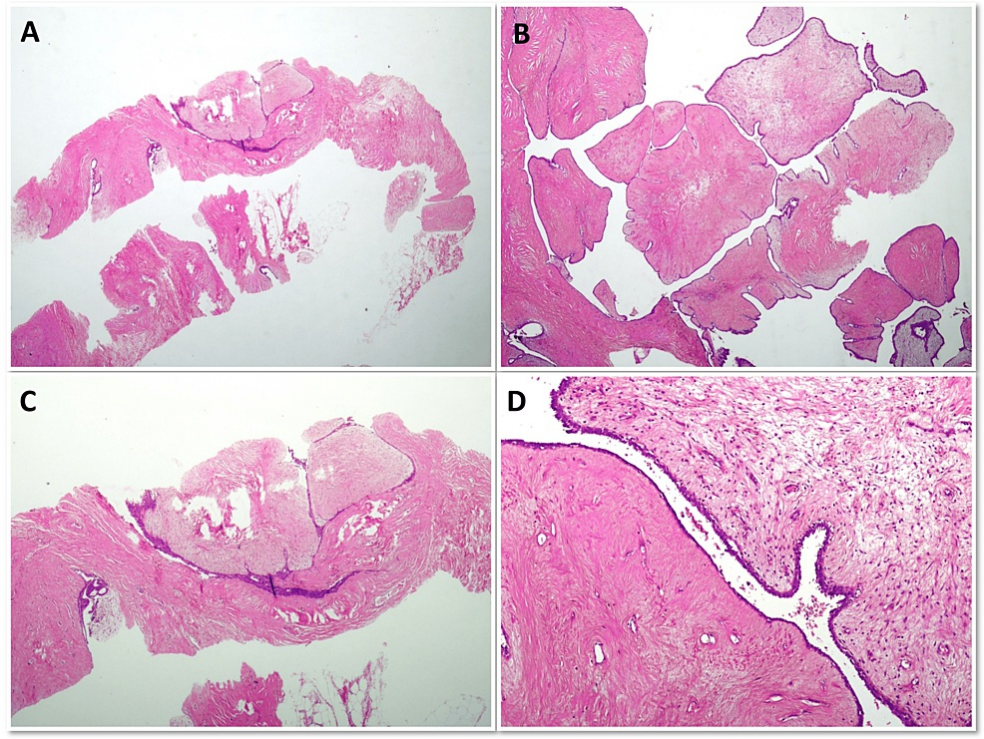
Benign phyllodes tumor diagnosed as fibroadenoma on CNB (Aand B) CNB showing stomal expansion but the stroma is hyalinized and the cellularity is low; (C&D) Excision specimen showing (C) leaf-like architecture and (D) stromal heterogeneity. CNB: Core needle biopsy

Among 125 cases characterized using CNB, the CNB- and subsequent surgical excision specimen-based diagnoses were concordant in 113 (90.4%) cases. Among 12 cases with discordant diagnoses, three (25%) cases of CNB-based FA diagnosis were diagnosed as PT on the excision specimen (Figure 1).

**Figure 2:**
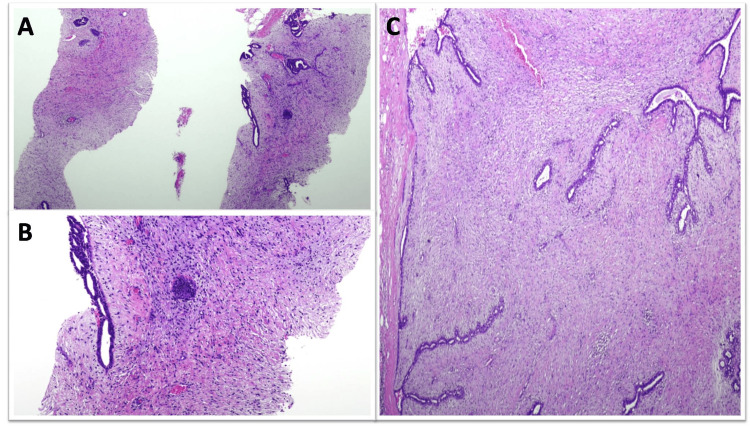
Cellular fibroadenoma diagnosed as benign phyllodes tumor on CNB (A and B) CNB showing stromal expansion and increased stromal cellularity; (C) Excision specimen showing an area of stromal expansion, increased stromal cellularity, and intracanalicular growth pattern. Other features of BPT were not present. CNB: Core needle biopsy

**Figure 3:**
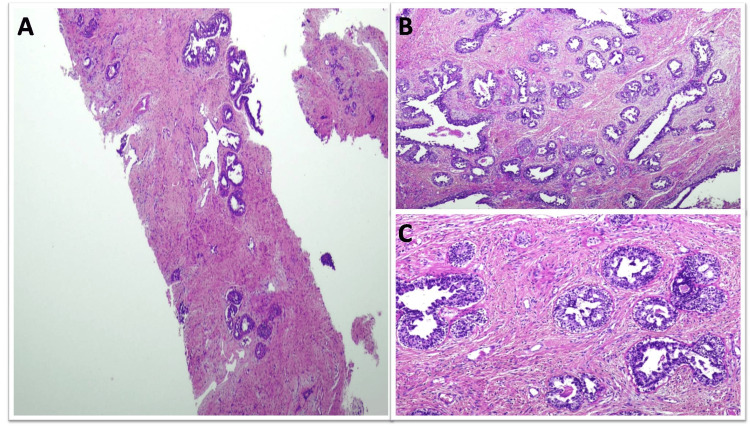
Juvenile fibroadenoma diagnosed as benign phyllodes tumor on CNB (A) CNB showing increased stromal cellularity; (B and C) Excision specimen showing cellular stroma with pericanalicular growth pattern. The epithelial component is showing prominent usual ductal hyperplasia. CNB: Core needle biopsy

Tumor sizes in these cases were 6 cm, 12.5 cm, and 17.5 cm. Two of these cases were benign PTs, and one case was borderline PT. Nine (75%) cases diagnosed as PT on CNB were diagnosed as FA on surgical excision specimen. Five of these FAs were conventional, two FAs were cellular (Figure 2),

One FA was complex and one FA was juvenile (Figure 3).

These tumors ranged in size from 1.8 to 6.5 cm with a median of 4.5 cm. All PT cases underreported as FA on CNB were >5 cm in size. Table 2 provides a summary of the age and tumor size of cases with discordant diagnoses on CNB and surgical excision specimens.

**Table 2:** Summary of discordant diagnosis on CNB and excisional biopsies. (n=12) CNB: Core needle biopsy

Serial #	Age (Years)	Tumor size (cm)	CNB diagnosis	Surgical excision diagnosis
1	23	17.5	Fibroadenoma	Benign phyllodes tumor
2	32	6	Fibroadenoma	Benign phyllodes tumor
3	38	12.5	Fibroadenoma	Borderline phyllodes tumor
4	29	5.8	Phyllodes tumor	Cellular fibroadenoma
5	46	6.5	Phyllodes tumor	Cellular fibroadenoma
6	57	1.8	Phyllodes tumor	Conventional fibroadenoma
7	23	5.7	Phyllodes tumor	Juvenile fibroadenoma
8	20	5	Phyllodes tumor	Complex fibroadenoma
9	18	2.4	Benign phyllodes tumor	Conventional fibroadenoma
10	32	3.7	Benign phyllodes tumor	Conventional fibroadenoma
11	33	4.5	Benign phyllodes tumor	Conventional fibroadenoma
12	46	4.5	Benign phyllodes tumor	Conventional fibroadenoma

In 23 cases, PTs were further categorized into benign, borderline, and malignant categories on CNB. One out of eight benign PTs on CNB was upgraded to borderline PT on the excision specimen. One out of 14 borderline PTs on CNB was upgraded to malignant PT, and another case was downgraded to benign PT on the excision specimen. One case diagnosed as malignant PT on CNB remained malignant on the excision specimen. Table 3 demonstrates the correlation between PT categories on CNB and those on surgical excision specimens.

**Table 3:** Correlation between the phyllodes tumor categories on CNB and surgical excision specimen. (n=40)* *Cases in which the diagnosis of phyllodes tumor was committed on CNB CNB: Core needle biopsy

CNB diagnosis	Surgical excision diagnosis		
	Benign (n=20)	Borderline (n=17)	Malignant (n=3)
Benign (n=8)	7 (87.5%)	1 (12.5%)	0
Borderline (n=14)	1 (7.1%)	12 (85.7%)	1 (7.1%)
Malignant (n=1)	0	0	1 (100%)
Uncategorized (n=17)	12 (70.6%)	4 (23.5%)	1 (5.9%)

Two cases diagnosed as conventional FA on CNB turned out to be complex FA on the excision specimen. One case of juvenile FA and one case of complex FA diagnosed on CNB were also diagnosed as the same on the excision specimen.

In 125 cases for which a definitive diagnosis was committed based on CNB, the diagnostic accuracy of CNB-based FEL diagnosis was 90.4%.

## Discussion

FELs are biphasic neoplasms of the breast including FA and PT. FA is the most common breast neoplasm comprising 20%-50% of all breast biopsy specimens, while PT comprises 0.3%-1% of all breast neoplasms and 2.5% of FELs [9,10]. These tumors differ in their biological behavior and management [1,2,9]. Researchers found marked interobserver variability and a low level of agreement among pathologists for the diagnosis of FELs on CNB [1,11]. The difficulty in distinguishing between FA and BPT is attributed to overlapping histological features, lack of defined cut-off values, heterogeneity of histological features within the same tumor, and inability to apply histological criteria on limited CNB material [4-6,12]. The positive predictive value (PPV) for FEL was 1.9% compared to an overall PPV of 25.6% in breast imaging-reporting and data system (BI-RADS) category 3 lesions [3]. A PPV of CNB-based PT diagnosis ranged from 12.5% to 70% [1,5,6,12].

Histological features, such as the growth pattern, stromal overgrowth, and tumor margins, can be optimally assessed on excision specimen specimens. Additionally, the assessment of mitotic counts, atypia, and stromal heterogeneity can be compromised due to the limited amount of CNB material [1]. According to Jacobs et al., extremes of stromal cellularity (mild vs. marked) are useful in differentiating FA from PT. However, moderate cellularity, mitotic count of 2-3/10 high-power fields (HPFs), and atypia are non-specific features. Furthermore, stromal heterogeneity and subepithelial condensation are prone to the subjectivity of histopathologists [6]. Moreover, Lee et al. showed that increased stromal cellularity, stromal overgrowth, CNB fragmentation, and adipose tissue within the stroma are useful histological features of FEL on CNB. These features were more frequently observed in PT than in FA with good interobserver agreement [12].

Bandyopadhyay et al. adopted the terminology of FEL for lesions with cellular stroma and ≤2 mitoses/10 HPFs [1]. They assessed intra- and interobserver variability among five histopathologists for diagnosing 50 FEL cases on CNB in three rounds conducted 2 weeks apart. In the 1st round, the histopathologists were not provided with any histological criteria. In the 2nd round, the list of histological criteria was provided to the histopathologists to establish the diagnosis. In the 3rd round, the histological criteria were defined. Excision specimen-based diagnosis was considered the gold standard. The mean accuracy rate in each round was calculated and significantly improved from 40% to 48% and then to 67% from the 1st to the 3rd round. The accuracy was higher for the FA group than for the PT group. Fair interobserver agreement was evident in all 3 rounds, which also insignificantly improved during these rounds. Among histological features, the poorest agreement was observed for the mitotic count, whereas the best agreement was reached for stromal fragmentation [1]. PT cases that were underdiagnosed as FA on CNB showed mild stromal cellularity without increased mitotic activity and atypia. Age of these patients ranged from 29 to 43 years. One of the FA cases, which was overdiagnosed as PT on CNB, showed moderate cellular stroma, stromal heterogeneity, and fragmentation. The patient’s age was 37 years [1]. Hence, the diagnostic accuracy of CNB could improve by listing and appropriately defining the standardized diagnostic histological criteria [1].

Juvenile FA (JFA) and cellular FA (CFA) are two FA variants that are especially difficult to differentiate from BPT on CNB. These variants even pose diagnostic challenges on excision specimens [9,13]. JFA shows increased stromal cellularity and mitotic activity, making its differentiation from BPT difficult. JFA typically exhibits a pericanalicular growth pattern contrasting with exuberant intracanalicular growth seen in PT [9]. In our study, three cases were diagnosed as JFA on the excision specimen. On CNB, one of these cases was diagnosed as PT, one case was diagnosed as FEL, and one case was diagnosed as JFA.

The cases diagnosed as CFA exhibit increased stromal cellularity and intracanalicular growth pattern; therefore, these can resemble BPT [9]. The distinction between CFA and BPT is subjected to marked interobserver variability, even among pathologists specialized in breast pathology [11]. CFA is also associated with local recurrence [14]. In a study of 90 cellular FELs (CFELs), the cases were divided based on five histological features (mitotic activity, stromal overgrowth, stromal cell atypia, intracanalicular growth, and invasive growth) into three groups: BPT (showing four or five features), CFA (showing one or two features), and CEFL of intermediate type (showing three features). A follow-up revealed local recurrence in 14.3% of CFA, 3% of intermediate-type CEFL, and no recurrence in the BPT group [14].

Complex FA is another histological FA variant characterized by the presence of ≥1 of the following histologic features: sclerosing adenosis, papillary apocrine metaplasia, epithelial calcification, and cysts ≥3 mm in diameter [15]. In our study, five cases were diagnosed as complex FA on the excision specimen. On CNB, two of these cases were diagnosed as conventional FA, one was diagnosed as PT, one was diagnosed as complex FA, and one was diagnosed as a descriptive diagnosis. Both cellular FAs in our study were overdiagnosed as PT on CNB.

PT more frequently exhibits hyperplastic changes in its benign epithelial component compared to FA. Higher stromal cellularity, high stroma-to-epithelium ratio, stromal atypia, increased mitoses, presence of adipose tissue within the stroma, and fragmentation of the core are features favoring the diagnosis of PT on CNB. On CNB of PT cases, the stromal expansion might not reach the level of “stromal overgrowth” but is still helpful in favoring the diagnosis of PT over FA. Furthermore, the presence of adipose tissue and multinucleated giant cells in the stroma is more common in PT than in FA [9].

PT generally presents at an older age and with a larger size than FA [16]. However, in a study of 36 FAs and 14 PTs, patient’s age in both groups overlapped. The patient’s age range was 20-61 years (median: 41 years) in the FA group and 28-66 years (median: 41 years) in the PT group [1]. Tumor size was 1-3.5 cm (median: 1.8 cm) in the FA group and 1.5-22 cm (median: 3.5 cm) in the PT group. More than one-third of PTs were <4 cm. The smaller tumor size of PT can be attributed to the current rise in mammographic screening programs, resulting in the early detection of breast tumors [1]. In a comparative study of 48 FEL cases diagnosed on CNB followed by surgical excision specimen, the patient’s age and tumor size did not significantly differ between FA and PT groups [17]. We observed that FA and PT age ranges overlap; however, the mean age of the FA group was lower than the mean age of the PT group (30.7 vs. 40.46 years). In our study, the tumor size range (1.1-10 cm) and mean size (4.4 cm) in the FA group were lower than the tumor size range (1-32 cm) and mean size (7 cm) in the PT group. All three PT cases under-reported as FA on CNB were >5 cm in size.

A recent comprehensive review of histological FEL features on CNB has stated that there is no single diagnostic histologic feature to differentiate between these two entities; hence, the diagnosis should be based on a constellation of multiple histological features. In challenging scenarios, the diagnosis of “benign FEL” could be established, followed by a definitive characterization on the excision specimen [9]. In cases diagnosed with FEL on CNB, the percentage of PT diagnoses on excision specimens is 18%-42%. The majority of these PTs belong to the benign category, and only a few cases belong to the borderline category [18]. Among 41 cases diagnosed as FEL on CNB, 13 (39%) cases were diagnosed as PT on the excision specimen.

Immunohistochemistry is also not helpful for diagnosis because the expression of CD34, proliferating cell nuclear antigen, and Ki-67 does not differ between FA and PT [18,19]. Furthermore, these tumors harbor similar gene mutations but at considerably different frequencies. TERT and RARA mutations are more common in PT than in FA (50% vs. 7% and 41% vs. 14%, respectively), while MED12 mutations are more frequent in FA [8]. The diagnostic utility of these molecular tests is limited due to cost-effectiveness. Moreover, PT cases more frequently show heterogeneous architecture and higher BI-RAD category (4b or greater) and less frequently show internal vascularity on imaging [17]. 

Assessing the histologic grade/category of PT is important since borderline and malignant PTs are associated with increased local recurrence and metastatic potential. However, further categorization is impossible on CNB because histological features, such as tumor margins and stromal overgrowth, cannot be assessed using limited CNB material [9]. Tsang et al. found that stromal cellularity, mitotic count, and pleomorphism on CNB correlate well with the PT category on excision specimen [7]. The definitive characterization of PT requires examination of the entire lesion. Thus, considering the limitations of CNB, characterization is usually done on excision specimen [9]. In our study, PTs were further categorized on CNB in 23 out of 40 cases. Of these, three cases were inaccurately categorized (Table 3).

FA and PT treatment differs considerably as FAs are treated conservatively or by enucleation, while PTs require excision with clear wide margins [9,20,21]. Some authors suggest that BPT can be treated with marginal excision due to a low recurrence rate [14,17].

The main limitations of this study include the single-center design and small sample size. A multicenter study would increase the sample size and add the experience of more histopathologists. Our study did not assess the interobserver variability, which had been assessed in other studies on the accuracy of CNB-based diagnosis of FEL.

## Conclusions

Our study showed good accuracy of CNB-based diagnosis. The likelihood of PT increases with increasing tumor size. The possibility of PT in a tumor >5 cm in size should always be considered, with increased caution for tumors >10 cm. CFA, JFA, and complex FA can impose diagnostic challenges for histopathologists on CNB, thereby being misdiagnosed as PT. The diagnostic accuracy of CNB can be enhanced by increasing the number of cores, correlation with radiological findings, and clinical correlation with the treating surgeon. Moreover, surgical excision should be considered for lesions in which CNB findings are discordant with clinical or radiologic findings.
